# The moderating role of recreational substance use in the association of Mediterranean diet with academic performance among adolescents

**DOI:** 10.1038/s41598-023-37529-8

**Published:** 2023-07-04

**Authors:** José Francisco López-Gil, Lee Smith, Anelise Reis Gaya, Desirée Victoria-Montesinos, Héctor Gutiérrez-Espinoza, Eva Herrera-Gutiérrez, Antonio García-Hermoso

**Affiliations:** 1 Navarrabiomed, Hospital Universitario de Navarra, Universidad Pública de Navarra (UPNA), IdiSNA, Pamplona, Navarra Spain; 2 Department of Environmental Health, Harvard University T.H. Chan School of Public Health, Boston, MA USA; 3 One Health Research Group, Universidad de Las Américas, Quito, Ecuador; 4 Centre for Health, Performance and Wellbeing, Anglia Ruskin University, Cambridge, UK; 5 School of Physical Education, Physiotherapy and Dance, Post-Graduate Program in Human Movement Sciences, Federal University of Rio Grande Do Sul, Porto Alegre, Brazil; 6 Faculty of Pharmacy and Nutrition, UCAM Universidad Católica San Antonio de Murcia, 30107 Murcia, Spain; 7 Escuela de Fisioterapia, Universidad de las Américas, 170504 Quito, Ecuador; 8 Department of Developmental and Educational Psychology, Faculty of Psychology, Espinardo Campus, University of Murcia, Murcia, Spain

**Keywords:** Health care, Risk factors

## Abstract

No study has examined the potential moderating role of recreational substance use in the relationship between the Mediterranean diet (MedDiet) and academic performance. The aim of this study was to test the potential moderating role of recreational substance use (i.e., alcohol, tobacco, and cannabis) in the association of adherence to the MedDiet with academic performance among adolescents. This cross-sectional study included a sample of 757 adolescents (55.6% girls) aged 12–17 years from the *Valle de Ricote* (Region of Murcia). The Region of Murcia is an autonomous community of Spain located in the southeast of the Iberian Peninsula, along the coast of the Mediterranean Sea. Adherence to the MedDiet was assessed by the Mediterranean Diet Quality Index for Children and Teenagers (KIDMED). Recreational substance use (i.e., tobacco, alcohol, cannabis) was self-reported by adolescents. Academic performance was assessed by the school records at the end of the academic year. The relationship between adherence to the MedDiet and academic performance was moderated by both tobacco and alcohol use (for grade point average and all school records). In conclusion, higher adherence to the MedDiet was related to greater academic performance in adolescents, but recreational substance use could moderate this association.

## Introduction

Adolescence is accompanied by significant physical, cognitive, and psychosocial changes that influence interactions, decision-making processes, thoughts, and feelings^1^. More specifically, adolescence is a critical stage for brain maturation, owing to synaptic pruning, myelination, and neural connection development, especially in the prefrontal cortex^2,3^. This brain maturation is accompanied by the development of progressively more complex cognitive skills, which, in turn, are bidirectionally related to academic performance^4^.

Academic performance is crucial for adolescents because it plays a significant role in their overall development and future prospects. For instance, academic performance could determine an individual's future academic career and job opportunities^5^. Similarly, engaging in learning activities, studying various subjects, and acquiring knowledge enhances critical thinking skills, problem-solving abilities, and intellectual capacity^6^. Achieving academic goals fosters a sense of accomplishment, self-esteem, and confidence^7^, which can contribute to better mental health outcomes^8^. Despite these benefits, academic performance may be affected by numerous interpersonal (i.e., socioeconomic status^9^), intrapersonal (i.e., sleep^10^, physical activity^11,12^, sedentary behavior^11^, obesity^13^), and institutional factors (i.e., type of school^14^).

The literature has reported that adolescents are highly prone to addiction compared to other age groups^15^. During this life phase, there is a strong inclination toward experimentation, curiosity, susceptibility to peer pressure, rebellion against authority, and poor self-worth, which makes such individuals vulnerable to substance abuse^16^. Multiple adverse childhood experiences (e.g., recreational substance use) are a major risk factor for many later-life mental health complications^17^. Regarding recreational substance use, tobacco, alcohol, and cannabis are substances most commonly used by adolescents^18^. In this sense, smoking, excessive drinking, or cannabis use during adolescence have both immediate and long-term health consequences^19–21^. Smoking^22^, alcohol use^23^, or cannabis use^23,24^ in adolescents may harm the developing brain. Similarly, recreational substance use and academic performance are likely to be inversely associated (i.e., higher alcohol use and smoking are both associated with lower academic performance)^25^.

Importantly, diet is a modifiable lifestyle behavior that can influence brain maturation and, consequently, cognition and academic performance^26^. In this regard, a systematic review showed moderate relationships of higher overall diet quality and healthier eating habits with greater academic performance, such as regular breakfast consumption and lower consumption of energy-dense foods and foods with low nutritional value^27^. Concerning healthy dietary patterns, following a Mediterranean diet (MedDiet) has been shown to be beneficial in improving cognitive function (e.g., memory, executive function, visual constructs)^28^ and cognitive performance^29^. This fact could be related to the specific micronutrients provided by this diet (e.g., vitamin C, vitamin E, folate) of its particular foods, including for example, olive oil, fruits, vegetables, legumes, cereals, nuts, or dairy products^30^.

Some studies have previously analyzed the relationship between the MedDiet and academic performance among young people^13,31,32^, indicating that higher adherence to the MedDiet was associated with greater academic performance. However, studies examining the presence of third variables in this relationship are scarce^13^. For instance, Tapia-Serrano et al.^13^ found that body mass index mediates the relationship between adherence to the MedDiet and academic performance. Moreover, Adelantado-Renau et al.^31^ reported that this relationship was mediated by sleep duration.

While there may not be specific studies examining the direct moderation effect of recreational substance use on the relationship between MedDiet and academic performance in adolescents, it is reasonable to assume that recreational substance use could potentially diminish the effects of a healthy diet on academic performance outcomes. The detrimental effects of substance use on cognitive function and academic performance might counteract the potential benefits derived from following a MedDiet. Given the above, a deeper understanding of the role of recreational substance use in the relationship between MedDiet and academic performance could be useful for parents/guardians, public health researchers, and policymakers. Therefore, the aim of this study was to test the potential moderating role of recreational substance use (i.e., alcohol, tobacco, and cannabis) in the association of adherence to the MedDiet with academic performance among adolescents.

## Material and methods

### Study design and population

This was a secondary cross-sectional analysis using data from the Eating Healthy and Daily Life Activities (EHDLA) research, which examined a representative sample of adolescents aged 12–17 years from *Valle de Ricote* (Region of Murcia, Spain). The Region of Murcia is an autonomous community of Spain located in the southeast of the Iberian Peninsula, along the coast of the Mediterranean Sea. The final sample involved 757 adolescents (55.6% girls). For this study, a total of three secondary schools (two public and one private with private funds) were recruited. The detailed methodology of the EHDLA study, which collected data during the 2021/2022 academic year, is published elsewhere^33^.

To participate in this study, parents/guardians of potentially participating adolescents were asked to provide written informed consent prior to adolescent enrollment. Furthermore, both parents/guardians and their children obtained an information sheet indicating the objectives of the EDHLA research project, as well as all the tests and questionnaires to be implemented. Moreover, participants were also asked to confirm their willingness to enroll in the study.

This study obtained ethics approval from the Bioethics Committee of the University of Murcia (ID 2218/2018) and the Ethics Committee of the Albacete University Hospital Complex and the Albacete Integrated Care Management (ID 2021-85). Furthermore, this study respected the human rights of the participants enrolled and followed the principles of the Helsinki Declaration.

### Variables

#### Adherence to the Mediterranean diet (independent variable)

Adherence to the MedDiet was assessed by the Mediterranean Diet Quality Index for Children and Teenagers (KIDMED), which has previously been validated in the young Spanish population^34^. The KIDMED includes a 16-question test and varies from − 4 to 12 points. Items reporting unhealthy aspects regarding the MedDiet were scored with − 1 point, and those reporting healthy aspects were scored with + 1 point. The sum of all scores from the KIDMED test was categorized into three different levels of adherence: (a) high MedDiet (> 8 points), (b) moderate MedDiet (from 4 to 7 points), and (c) low MedDiet (≤ 3 points).

#### Recreational substance use (moderator variable)

Tobacco, alcohol, and cannabis use were independently determined by the following question: “During the past 30 days, on how many days did you smoke cigarettes (and the same separate questions were asked for alcohol and cannabis use)?”. The available response options were as follows: (a) never, (b) once or twice, (c) 3–5 times, (d) 6–9 times, (e) 10–19 times, (f) 20–29 times, or (g) 30 times or more^35^. For further analyses, adolescents who reported a use from “once or twice” to “30 times or more” were considered “tobacco smokers/alcohol users/cannabis smokers”. Conversely, those reporting “never” were considered “nontobacco smokers/nonalcohol users/noncannabis smokers”.

#### Academic performance (dependent variable)

Academic performance was assessed by the school records (provided by each school center) at the end of the academic year and was determined in two different ways: (a) according to the grade point average (GPA), which includes grades obtained in language, math, and foreign language; and (b) as the average of the school records based on all the subjects attended by each adolescent, which varied from 9 to 11 subjects. This decision was based on the objective of including representativeness of all subjects taken by the adolescents. Further information about math, language, foreign language (individually), or math and language (combined) is shown in Tables S1, S2 and S3.

#### Covariates

Age and sex were self-reported by adolescents. Socioeconomic status was assessed with the Family Affluence Scale (FAS-III)^36^. The FAS-III score was calculated by the sum of the responses from six different items related to family (i.e., vehicles, bedrooms, computers, bathrooms, dishwashers, and travels). The final score ranged from 0 to 13 points. A higher score indicates greater socioeconomic status. Based on standard protocols, the body weight of the adolescents was determined by an electronic scale (with an accuracy of 0.1 kg) (Tanita BC-545, Tokyo, Japan), and the height was determined by a portable height rod with a precision of 0.1 cm (Leicester Tanita HR 001, Tokyo, Japan). Body mass index was computed by dividing body weight (in kg) by height (in m^2^).

The Youth Activity Profile Physical (YAP), a self-administered 7-day (previous week) recall questionnaire that contains 15 different items, was applied to determine physical activity and sedentary behavior among adolescents^37^. The Spanish version of YAP (YAP-S) has been validated and adapted previously^38^. The items include a 5-point Likert scale and are separated into three sections: activity at school, activity out-of-school, and sedentary habits. Physical activity (at school/out-of-school) and sedentary behavior (sedentary habits) scores were computed as the sum of the items in each section.

Sleep duration was evaluated by asking participants for weekdays and weekend days separately: “What time do you usually go to bed?” and “What time do you usually get up?”. The average daily sleep duration was computed for each participant as follows: [(average nocturnal sleep duration on weekdays × 5) + (average nocturnal sleep duration on weekends × 2)]/7.

Energy consumption was estimated by a self-administered food frequency questionnaire (FFQ), which includes 45 items separated into 12 different food groups and was previously validated among the Spanish population^39^.

The selection of all the included covariates was based on previous findings in the scientific literature^9–12,40^.

### Statistical analysis

Concerning descriptive data, categorical variables were expressed as numbers and percentages. Conversely, continuous variables were reported as the means and standard deviation (SD). Moderation analyses were conducted using PROCESS macro 4.3 in R (RStudio software version 4.2.2, R Group for Statistical). The PROCESS macro applies ordinary least squares (OLS) analysis to estimate moderation models (model 1 in PROCESS) using academic performance (i.e., GPA or all school records) as dependent variables (*Y*), adherence to the MedDiet (i.e., KIDMED score) as independent variable (*X*), and recreational substance use (i.e., tobacco, alcohol, or cannabis) as a moderator variable (*W*), with a bootstrapping resampling approach with 10,000 samples (to estimate standard errors and confidence intervals)^41^. The theorical model is represented as follows: *Y* = *iY* + *b*_1_*X* + *b*_2_*W* + *eY*, where *b*_1_ and *b*_2_ are estimated regression coefficients, *eY* is an estimation error, and *iY* is a regression intercept. This model estimates *Y* (outcome) from two priors *X* (independent variable) and *W* (moderator variable). Preliminary analyses showed no interaction when sex was included as a moderating variable (*p* > 0.05 in all cases). Therefore, we performed the analyses without dividing by boys and girls. All analyses were adjusted for age, sex, socioeconomic status, body mass index, physical activity, sedentary behavior, sleep duration, and recreational substance use (e.g., when examining alcohol use, analyses were further adjusted for tobacco and cannabis use).

## Results

Descriptive data of the study participants are reported in Table 1. The KIDMED score mean was 6.6 ± 2.5. During the last 30 days, 8.1%, 18.9%, and 2.4% of the adolescents reported tobacco, alcohol, and cannabis use, respectively. Concerning academic performance, the lowest mean was found for math (5.8 ± 2.5). Conversely, the highest mean was 6.7 ± 1.9 for all school records (i.e., the average of all subjects taken by the adolescents).

**Table 1:** Descriptive data of the analyzed sample (N = 757).

Variables	M ± SD
Recreational substance use	
Tobacco smoker, n (%)	61 (8.1)
Alcohol user, n (%)	143 (18.9)
Cannabis smoker, n (%)	18 (2.4)
Adherence to the MedDiet	
KIDMED (score)	6.6 ± 2.5
Low MedDiet, n (%)	86 (11.4)
Moderate MedDiet, n (%)	387 (51.1)
High MedDiet, n (%)	284 (37.5)
Academic performance	
Language (score)	6.5 ± 2.4
Math (score)	5.8 ± 2.5
Foreign language (score)	6.3 ± 2.3
Language/math (score)	6.2 ± 2.1
GPA (score)	6.2 ± 2.1
All school records (score)	6.7 ± 1.9
Covariates	
Age (years)	14.0 ± 1.5
Sex	
Boys	336 (44.4)
Girls	421 (55.6)
FAS-III (score)	8.2 ± 2.1
YAP-S physical activity (score)	2.6 ± 0.7
YAP-S sedentary behavior (score)	2.6 ± 0.6
Sleep duration (min)	493.2 ± 53.4
BMI (kg/m^2^)	22.7 ± 4.7
Energy consumption (kcal)	2975.6 ± 1901.0

Data are expressed as the mean (standard deviation). Otherwise, it is specified. *BMI*, body mass index, *FAS-III,* family affluence scale-III, *GPA*, grade point average, *KIDMED,* Mediterranean Diet Quality Index for children and teenagers, *MedDiet*, Mediterranean diet, *YAP-S*, Spanish Youth Active Profile.

**Figure 1:**
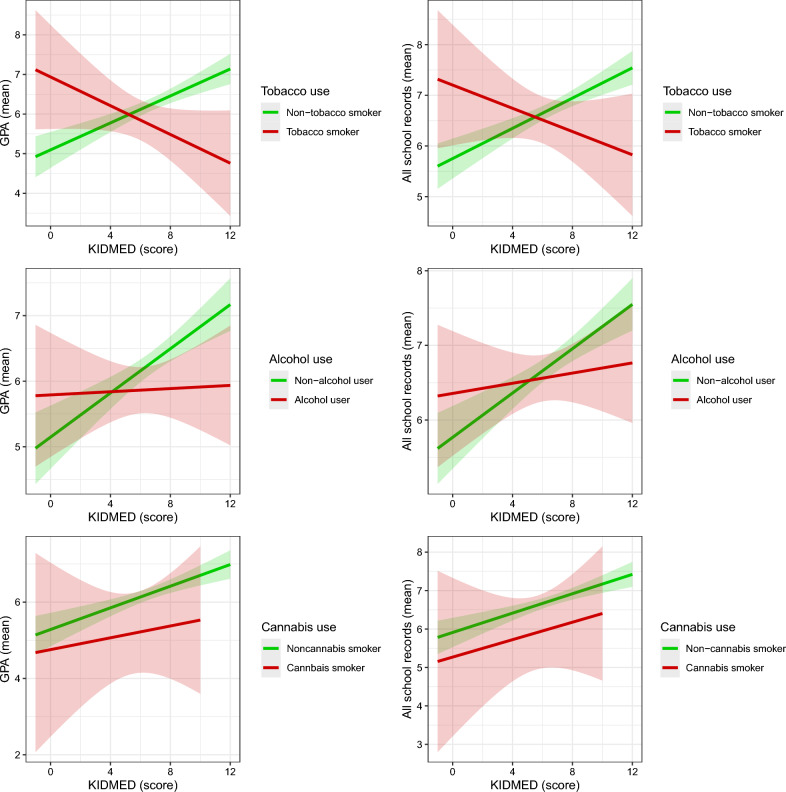
Moderating role of recreational substance use (i.e., tobacco, alcohol, and cannabis) in the relationship between adherence to the Mediterranean diet and the different academic performance variables. *GPA,* grade point average.

The moderating role of recreational substance use (tobacco, alcohol, or cannabis use) in the relationship between adherence to the MedDiet and the different academic performance outcomes is shown in Fig. 1. Supplementary Table S1 shows the specific values of the regression models examining the moderating role of recreational substance use (tobacco, alcohol, or cannabis) in the relationship between adherence to the MedDiet and all academic performance variables (i.e., GPA and all school records). The association between adherence to the MedDiet and academic performance was moderated by tobacco use (GPA: *B* = − 0.383, 95% CI − 0.606, − 0.164; all school records: *B* = − 0.287, 95% CI − 0.488, − 0.091). This moderating role was also observed for alcohol use (GPA: *B* = − 0.187, 95% CI − 0.348, − 0.029; all school records: *B* = − 0.138, 95% CI − 0.276, − 0.006).

## Discussion

### Main findings

Although our results showed that greater adherence to the MedDiet was associated with higher academic performance among adolescents, this association was only described in those adolescents who did not smoke tobacco and did not drink alcohol. Our findings showed that tobacco use had the strongest moderating effect for this relationship, even though the association between adherence to the MedDiet and academic achievement was larger for those who did not take substances (i.e., tobacco, alcohol, or cannabis) than for those who did. A previous systematic review by Burrows et al.^27^ underlined that some dietary patterns (e.g., regular breakfast consumption, lower consumption of energy-dense, nutrient-poor food, and overall diet quality) were relevant behaviors and modifiable aspects related to greater academic performance. In this sense, all these patterns are included in the KIDMED questionnaire, which assesses adherence to the MedDiet. More specifically, other studies have supported the association between adherence to the MedDiet and academic performance in Spain^13,31,32^, Greece^42^, Italy^43^, and Lebanon^44^. Notwithstanding, the aforementioned studies did not consider substance use, which, as our results indicate, could influence the results obtained. Although there are no previous studies examining the moderating role of recreational substance use in the relationship between adherence to the MedDiet and academic performance in adolescents, there are some possible mechanisms that could explain the findings.

### The moderating role of tobacco use status

We found that the association between adherence to the MedDiet and all academic performance indicators was moderated by smoking status. There are possible mechanisms that partially explain these findings. Nicotine, the main component of cigarettes, is an appetite suppressant that can reduce appetite and influence eating habits^45^. Supporting this notion, smokers have more frequent cravings for high-fat foods and consume more high-fat foods than nonsmokers^46^. Furthermore, it has been hypothesized that the effect of nicotine on smell and taste leads smokers to prefer meat and fat-rich foods instead of fruits or vegetables^47^. A higher consumption of fruits and vegetables (i.e., identity characteristics of the MedDiet) provides greater levels of vitamin C, which cooperates with vitamin E as an antioxidant^48^. Importantly, vitamin E has been related to higher executive function and academic performance in adolescents^49^. Moreover, smoking increases vitamin E requirements in humans, and it has been suggested to encourage smokers to stop smoking rather than to formulate dietary recommendations that will help them deal with the magnitude of oxidative stress experienced^50^. Similarly, smoking negatively affects preferences for vitamin C-rich foods (e.g., fruits, vegetables), and the inverse association between smoking and serum vitamin C levels occurs independently of dietary consumption^51^. Therefore, it may be speculated that smoking diminishes the beneficial association between the MedDiet and academic performance. Another possible reason is that heavy smoking (high nicotine content) increases arousal but impairs working memory capacity. Working memory is one of our core cognitive functions, allowing individuals to maintain information for shorter intervals of time and then work with that information^52^. It is possible that, despite the possible beneficial effect of the MedDiet on cognitive development (e.g., working memory)^53^, smoking could affect working memory capacity, which may lead to lower academic performance^52,54^.

### The moderating role of alcohol use status

In relation to alcohol use, our results indicate that the association between MedDiet and academic performance was moderated by the consumption of this substance for foreign language, GPA, and all school records. Although the interactions were not statistically significant in the other academic performance indicators, the association between MedDiet and academic performance was only significant for adolescents who did not consume alcohol in the last 30 days. There are some possible explanations justifying this finding. Adverse health behaviors such as risky drinking and unhealthy eating begin to cluster during adolescence^55^, and both have been demonstrated to track into and throughout adulthood^56^. Increased consumption of fruits and vegetables may also increase the intake of some micronutrients, such as folate, which has been linked with higher academic performance^57^. Conversely, chronic alcohol exposure (via ethanol content) may impair folate absorption by inhibiting expression of the reduced folate carrier and decreasing the hepatic uptake and renal conservation of circulating folate^58^. We speculate that the association between MedDiet and academic performance (via folate intake) may be lower in those who drink alcohol. Another possible explanation could lie in the role of ethanol oxidation in GABA and glutamate metabolism. Some foods characteristic of the MedDiet are a dietary source of GABA and glutamate^59^. In contrast, ethanol oxidation is particularly disruptive of glutamate and GABA metabolism and handling in the brain^60^. Since GABA and glutamate have a dissociable and dynamic role in predicting learning^61^, one possible hypothesis is that the potential beneficial effects of foods characteristic of the MedDiet (in relation to GABA and glutamate) are reduced when ethanol is ingested^60^. This fact could (at least in part) explain the lower academic performance.

### The moderating role of cannabis use status

Although lower academic performance was observed among cannabis smokers, the moderation analysis was not statistically significant in this case for any academic performance indicator. Possible explanations of this absence of significance could lie in the low prevalence of adolescent smoking cannabis or in the number of adolescents who might feel uncomfortable answering the question about cannabis use. It is possible that adolescents may perceive cannabis use as less socially acceptable than tobacco or alcohol use^62^. Therefore, caution is necessary when interpreting this result. Cannabis users have shown lower diet quality compared to never or previous users, particularly lower vegetables, greens and beans, total fruit, and whole fruit consumption^63^. Cannabis sativa has been documented to promote strong cravings for food and intensify the sensory and hedonic properties of food^64^. These effects, commonly referred to as "munchies", are attributed to the psychoactive compound tetrahydrocannabinol (THC) found in cannabis. THC could stimulate the release of hunger-inducing hormones and enhance the pleasure and reward aspects of eating, which may lead to a preference for high-calorie and low-nutrient foods^64^. We hypothesize that all these factors could produce a lower adherence to the MedDiet, which could lead to lower academic performance in adolescents. In addition, there are several undesirable effects of cannabis use among adolescents, such as problems with school and social life, difficulty thinking and problem solving, difficulty maintaining attention, or problems with memory and learning (among others)^24^. These factors have been observed to be associated with cognitive function^65^, as well as lower academic performance in adolescents^65,66^. Specifically, cannabis use has been linked with lower GPA, skipping classes, and longer time to graduate^67^ and thus likely influences academic performance. Furthermore, as previously mentioned for smoking, some MedDiet foods (e.g., fruits, vegetables) have an abundance of vitamins C and E, which have a concomitant role as antioxidants, and they seem to be related to academic performance^49^. Notwithstanding, although the impact of cannabis use on antioxidant vitamins is not well known, one previous study indicated a significant decrease in vitamin C and E levels in cannabis smokers compared with nonsmokers^68^. It is possible that cannabis use decreases these levels, which may lead to lower academic performance^49^.

### Methodological considerations

The results from the present study must be interpreted considering the study’s limitations. Because of the cross-sectional design of this study, causal inferences cannot be made. Future prospective observational and experimental studies are required to examine whether increased MedDiet or reduced recreational substance use leads to greater academic performance in adolescents. Likewise, information on MedDiet and recreational substance use through questionnaires may lead to some differential desirability bias because of information and recall bias. In addition, because of the low prevalence of participants reporting recreational substance use, a dose‒response relationship could not be established. Conversely, one strength of the study is that academic performance was objectively assessed and not self-reported by the adolescents^69^. Furthermore, we also examined overall academic performance, including all the subjects. Traditionally, studies of academic performance have focused on language arts, mathematics and English^13,32^. However, this could lead to a lack of representation of other disciplines. By focusing only on a few subjects, academic performance in other equally important subjects, such as science, history, art, or physical education, is ignored. Each of these subjects has its own set of skills and knowledge that are also valuable in assessing overall academic performance^70^. Another strength is that, to our knowledge, the results from this study offer (for the first time) cross-sectional evidence of the moderating role of recreational substance use in the relationship between MedDiet and academic performance among adolescents.

## Conclusion

Our study provides evidence that in Spanish adolescents, higher adherence to the MedDiet is related to greater academic performance, but recreational substance use could moderate this association. Hence, awareness-raising campaigns to prevent recreational substance use while promoting adherence to the MedDiet (e.g., by a multifactorial approach) could be relevant since it seems to be related to higher academic performance in adolescents. Because of the moderating effect of recreational substance use on the relationship between eating habits and academic performance, future studies in this area should include these variables, as failure to consider them could introduce bias into the results obtained.

## Supplementary Information

Supplementary Tables.

## Data Availability

The datasets used and/or analyzed during the current study will be available from the corresponding author on reasonable request.
