# Supplementary material for: The moderating role of recreational substance use in the association of Mediterranean diet with academic performance among adolescents
Source: Sci Rep. 2023 Jul 4;13:10816. doi: 10.1038/s41598-023-37529-8 (PMC10319864; doi:10.1038/s41598-023-37529-8)
Supplement: Supplementary file 1 — Supplementary Tables. [file 41598_2023_37529_MOESM1_ESM.docx]

**Table S1.** The moderating role of tobacco use in the relationship between the Mediterranean Diet Quality Index for Children and Teenagers score and academic performance among adolescents.

|  | Language | | Math | | Language/Math | | Foreign language | | GPA | | All school records | |
| --- | --- | --- | --- | --- | --- | --- | --- | --- | --- | --- | --- | --- |
| Predictors | *B* | 95% CI | *B* | 95% CI | *B* | 95% CI | *B* | 95% CI | *B* | 95% CI | *B* | 95% CI |
| Tobacco ^†^ |  |  |  |  |  |  |  |  |  |  |  |  |
| *MedDiet* ^‡^ *(score)* | **0.137** | **[0.066, 0.207]** | **0.185** | **[0.112, 0.257]** | **0.161** | **[0.094, 0.227]** | **0.162** | **[0.095, 0.228]** | **0.161** | **[0.099, 0.223]** | **0.141** | **[0.086, 0.194]** |
| *Tobacco use (yes)* | **2.029** | **[0.445, 3.784]** | **2.697** | **[0.845, 4.623]** | **2.368** | **[0.768, 4.081]** | **3.115** | **[1.508, 4.600]** | **2.617** | **[1.109, 4.135]** | **1.991** | **[0.672, 3.406]** |
| *Interaction* | **-0.287** | **[-0.555, -0.047]** | **-0.428** | **[-0.696, -0.155]** | **-0.358** | **[-0.603, -0.127]** | **-0.433** | **[-0.650, -0.206]** | **-0.383** | **[-0.606, -0.164]** | **-0.287** | **[-0.488, -0.092]** |

Data are expressed as nonstandardized values and 95% bootstrap confidence intervals using the percentile method. Bold indicates a significant association. Adjusted for age, sex, socioeconomic status, body mass index, physical activity, sedentary behavior, sleep duration, alcohol use, and cannabis use. MedDiet, Mediterranean diet. ^‡^ According to Mediterranean Diet Quality Index for Children and Teenagers (KIDMED) score.

**Table S2.** The moderating role of alcohol use in the relationship between the Mediterranean Diet Quality Index for Children and Teenagers score and academic performance among adolescents.

|  | Language | | Math | | Language/Math | | Foreign language | | GPA | | All school records | |
| --- | --- | --- | --- | --- | --- | --- | --- | --- | --- | --- | --- | --- |
| Predictors | *B* | 95% CI | *B* | 95% CI | *B* | 95% CI | *B* | 95% CI | *B* | 95% CI | *B* | 95% CI |
| Alcohol ^†^ |  |  |  |  |  |  |  |  |  |  |  |  |
| *MedDiet* ^‡^ *(score)* | **0.136** | **[0.062, 0.212]** | **0.181** | **[0.099, 0.264]** | **0.158** | **[0.088, 0.232]** | **0.178** | **[0.108, 0.249]** | **0.165** | **[0.099, 0.233]** | **0.144** | **[0.086, 0.202]** |
| *Alcohol use (yes)* | 0.575 | [-0.577, 1.787] | 1.106 | [-0.186, 2.398] | 0.840 | [-0.305, 2.051] | **1.400** | **[0.202, 2.586]** | 1.028 | [-0.060, 2.183] | 0.932 | [-0.009, 1.926] |
| *Interaction* | -0.119 | [-0.293, 0.044] | -0.167 | [-0.349, 0.015] | -0.143 | [-0.315, 0.022] | **-0.274** | **[-0.441, -0.103]** | **-0.187** | **[-0.348, -0.029]** | **-0.138** | **[-0.276, -0.006]** |

Data are expressed as nonstandardized values and 95% bootstrap confidence intervals using the percentile method. Bold indicates a significant association. Adjusted for age, sex, socioeconomic status, body mass index, physical activity, sedentary behavior, sleep duration, tobacco use, and cannabis use. MedDiet, Mediterranean diet. ^‡^ According to Mediterranean Diet Quality Index for Children and Teenagers (KIDMED) score.

**Table S3.** The moderating role of cannabis use in the relationship between the Mediterranean Diet Quality Index for Children and Teenagers score and academic performance among adolescents.

|  | Language | | Math | | Language/Math | | Foreign language | | GPA | | All school records | |
| --- | --- | --- | --- | --- | --- | --- | --- | --- | --- | --- | --- | --- |
| Predictors | *B* | 95% CI | *B* | 95% CI | *B* | 95% CI | *B* | 95% CI | *B* | 95% CI | *B* | 95% CI |
| Cannabis ^†^ |  |  |  |  |  |  |  |  |  |  |  |  |
| *MedDiet* ^‡^ *(score)* | **0.114** | **[0.046, 0.183]** | **0.152** | **[0.080, 0.225]** | **0.133** | **[0.067, 0.199]** | **0.127** | **[0.060, 0.194]** | **0.131** | **[0.070, 0.193]** | **0.117** | **[0.063, 0.170]** |
| *Cannabis use (yes)* | -0.277 | [-3.674, 1.975] | -0.665 | [-3.114, 1.300] | -0.471 | [-3.228, 1.423] | -1.034 | [-4.191, 0.986] | -0.659 | [-3.492, 1.173] | -1.002 | [-3.876, 0.760] |
| *Interaction* | 0.006 | [-0.369, 0.497] | -0.049 | [-0.376, 0.370] | -0.021 | [-0.351, 0.411] | 0.017 | [-0.375, 0.553] | -0.008 | [-0.345, 0.443] | 0.056 | [-0.245, 0.495] |

Data are expressed as nonstandardized values and 95% bootstrap confidence intervals using the percentile method. Bold indicates a significant association. Adjusted for age, sex, socioeconomic status, body mass index, physical activity, sedentary behavior, sleep duration, smoke use, and alcohol use. MedDiet, Mediterranean diet. ^‡^ According to Mediterranean Diet Quality Index for Children and Teenagers (KIDMED) score.
